# Electrospun Polycaprolactone Scaffolds with Marine-Derived Biosilica Nanoparticles (*Dragmacidon reticulatum*) for Bone Tissue Engineering Applications

**DOI:** 10.1021/acsomega.5c07836

**Published:** 2025-11-10

**Authors:** Giovanna do Espirito Santo, Julieta Leticia Merlo, Pablo Botta, Ana Cláudia Muniz Rennó, Guadalupe Rivero

**Affiliations:** † Department of Biosciences, Federal University of São Paulo (UNIFESP), 136 Silva Jardim Street, Santos, SP 11015-020, Brazil; ‡ Applied Electrochemistry Area, Materials Science and Technology Research Institute (INTEMA), CONICET-University of Mar del Plata, Colon 10850, 7600 Mar del Plata, Argentina; § Ceramics Area, INTEMA, CONICET-University of Mar del Plata, Colon 10850, 7600 Mar del Plata, Argentina; ∥ Biomedical Polymers Area, INTEMA, CONICET-University of Mar del Plata, Colon 10850, 7600 Mar del Plata, Argentina

## Abstract

Critical bone fractures require biomaterials capable of promoting efficient regeneration, with polymeric scaffolds with a bioactive phase being a promising approach. This study investigated the effect of the incorporation of different amounts (10, 15, and 20% w/w) of biosilica nanoparticles (nBS) extracted from the sponge *Dragmacidon reticulatum* within biodegradable polymer matrices based on poly­(ε-caprolactone) (PCL), with and without alkaline surface modification to increase polarity. Composites were processed by electrospun and casting, and the effect of the interconnected porosity on the structure, surface functionalization, and bioactivity was evaluated by comparison with the nonporous films’ counterparts. Electrospinning generated nanofibrous membranes with better nBS dispersion, while the films exhibited filler agglomeration. SEM-EDS analyses revealed the best homogeneous distribution of Si and Ca in the surface-modified nanofibers containing 15% nBS. Contact angle measurements, X-ray diffraction, and thermogravimetric analysis revealed that the morphological modifications and property enhancements achieved both through biosilica particle addition and surface modification impacted the films and electrospun membranes differently. Energy dispersive spectroscopy analysis after 21 days of immersion in simulated body fluid evidenced an intense apatite deposition on the nanofibrous membranes, while maintaining an interconnected porosity. On the other side, the films with higher biosilica content exhibited mineral deposits with a Ca/P ratio ≈ 1.64, similar to that of natural hydroxyapatite. However, the Ca^2+^ release estimation by X-ray fluorescence indicated that a submicrometer porous architecture with large surface area favors more dynamic mineralization and deposition processes in time. Finally, tests with pre-osteoblasts confirmed cytocompatibility of the surface-modified electrospun composites with the most promising behavior. It is concluded that the compositional design combined with electrospun and surface modification resulted in scaffolds with biomimetic microarchitecture, better nBS dispersion, effective surface functionalization, and superior bioactive response to the films. This strategy represents an advance in the engineering of biomaterials aimed at critical bone regeneration.

## Graphical abstract

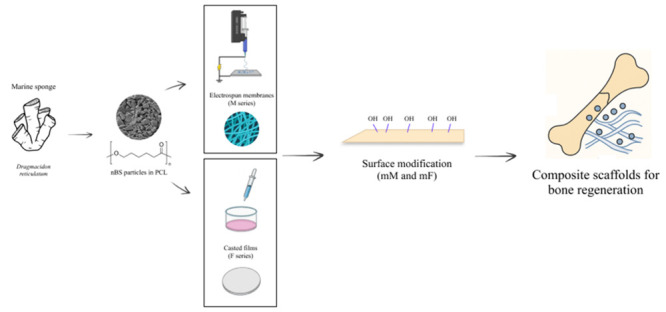

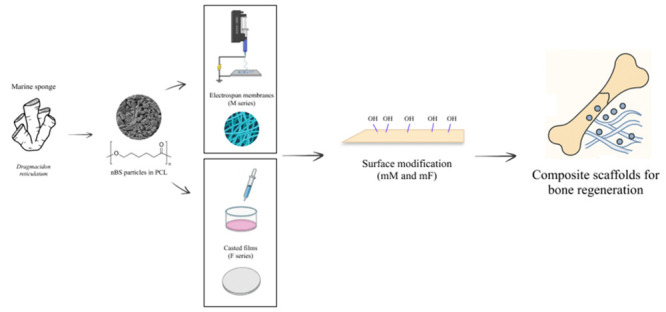

## Introduction

Bone fractures remain a major public health issue worldwide with growing socioeconomic impacts, particularly in aging populations. In Latin America alone, the total cost of fracture treatment reached approximately USD 1.17 billion in 2018.^1^ In this context, strategies that accelerate bone repair are essential to reduce hospitalization time and prevent complications such as pseudarthrosis and infections, thereby lowering healthcare costs and improving patients’ quality of life.

Over the past decades, various bioactive and biodegradable materials have been investigated for applications in bone regeneration. Among these, biosilica (BS) has attracted increasing attention due to its unique physicochemical and biological characteristics. This natural form of silica presents a high degree of organization and surface reactivity, which contributes to its biological performance. BS is a structured material produced by marine sponges through a mild enzymatic process mediated by silicatein, which allows silica condensation under physiological conditions.^2^ BS is considered a promising biocompatible material for bone regeneration due to its osteoinductive properties, promoting mineralization and bone cell differentiation.^3^ It has been shown to induce hydroxyapatite formation and stimulate the expression of osteoprotegerin in osteoblast-like cells.^4^ The morphogenetic activity of BS on human bone cells further highlights its potential for bone repair and regeneration.^5^ Compared to synthetic bioactive glasses, BS offers several advantages, including lower cost, intrinsic biocompatibility, and the capacity to release orthosilicic acid, which stimulates osteoblast activity and collagen synthesis.^6,7^ Regarding the potential of BS for bone regeneration, Gabbai-Armelin et al. (2019)^8^ reported that BS, extracted from the marine sponge *Dragmacidon reticulatum*, presents structural similarity with the natural extracellular matrix of bone, as well as more controlled calcium uptake and release and greater cell viability and proliferation than bioglass-based counterparts.

To exploit its regenerative properties, several studies have focused on incorporating BS into polymeric matrices to produce composite scaffolds for bone tissue engineering. One explored strategy involves the enzymatic formation of silica within polymeric structures, as demonstrated by Müller et al.,^9^ resulting in an active matrix that allows for the growth and mineralization of osteoblastic cells. However, this approach has limitations, such as a low silica yield and negligible differences between modified and unmodified scaffolds, which reduce its practical applicability.

As an alternative, the direct incorporation of BS particles as fillers into biodegradable polymer matrices offers more control over the composition and performance. For example, Wu et al. (2020)^10^ purified and heat-treated exactinellid sponge spicules to obtain 10–40 nm siliceous particles via wet milling, which were incorporated at 1, 3, and 5 wt % into a polylactic acid (PLA) matrix. The resulting electrospun composite fibers were cytocompatible with CCD-966SK cells. Müller et al. (2014)^9^ applied the *in situ* BS formation strategy by incubating electrospun poly­(ε-caprolactone) (PCL) mats containing tetraethyl orthosilicate (TEOS) with the silicatein enzyme. While the resulting BS coating provided a bioactive matrix that supported osteoblast-related SaOS-2 cell mineralization, the actual silica content remained low, and both coated and uncoated fibers exhibited similar biological responses in osteogenic media.

PCL has been frequently chosen as a biodegradable scaffold matrix for biomedical applications, given its slower degradation rate than PLA, which does not produce a localized acidic environment. However, its hydrophobic nature may limit cell adhesion and proliferation. To address this limitation, alkaline hydrolysis has been often employed as a surface modification strategy to increase surface polarity and enhance biointegration.^8^

The biofabrication potential of silicatein has also been explored for the development of BS-based 3D scaffolds through printing and electrospun techniques to meet porosity requirements.^4^ Among them, electrospinning stands out as a simple and highly versatile technique for the production of submicrometer fibrous mats with interconnected porosity, large surface area, and biomimetics of the extracellular matrix. It allows the fabrication of scaffolds with tunable mechanical, structural, and biochemical properties by adjusting parameters such as the polymer concentration, voltage, and collector design. Furthermore, this technique allows the incorporation of a wide range of active agents, excipients, or pharmaceutical ingredients into the fibers, either by direct mixing, coaxial configurations, or postprocessing surface functionalization, for a broad spectrum of biomedical applications.^5^ As detailed by Rivero et al. (2019),^6^ electrospun nanofibers have been successfully employed as scaffolds for tissue engineering, drug delivery systems, wound dressings, and biosensors, due to their hierarchical architecture, functional adaptability, and ability to faithfully replicate the native extracellular matrix. In particular, ceramic fillers such as BS can be directly integrated into the polymer solution, resulting in composite nanofibers with enhanced properties for bone tissue engineering. The incorporation of inorganic bioactive phases such as BS into electrospun PCL fibers can further enhance the osteoinductive and mechanical characteristics of the scaffold.^6^

Despite these promising findings, there is still limited knowledge about how the nBS concentration and the processing method used to produce composite scaffolds influence their physicochemical and biological properties. Most previous studies have employed low filler contents or focused on a single fabrication technique, limiting our understanding of how these variables affect the scaffold performance.

In this work, nBS extracted from the marine sponge *Dragmacidon reticulatum* was incorporated into PCL at three different concentrations (10, 15, and 20 wt %) to fabricate electrospun composite membranes. For comparison, solvent-cast films with identical compositions were prepared as nonporous counterparts. Furthermore, the groups subjected to alkaline hydrolysis to increase surface hydrophilicity were separated, and the resulting morphological changes were carefully evaluated compared to those of the unmodified groups. Therefore, the filler content and processing method were systematically studied. This study aims to clarify their combined effects on the morphological, physicochemical, thermal, and biological properties of PCL/nBS composites. The results are expected to contribute to the rational design of advanced biomaterials for bone tissue regeneration.

## Materials and Methods

### Marine Sponges and BS Extraction

BS was extracted from the marine sponge species *Dragmacidon reticulatum*, collected in Praia Grande, São Sebastião, Brazil, under authorization of the Sistema Nacional de Gestão do Patrimônio Genético e do Conhecimento Tradicional Associado (SISGen) and by the Sistema de Autorização e Informação em Biodiversidade (SISBio), granted under codes A33FAC6 and 289171, respectively.

The extraction protocol follows Weaver et al. (2003).^21^ Initially, the marine sponges were thoroughly rinsed with distilled water to remove any residual impurities. They were then sectioned into smaller fragments (approximately 1 × 1 cm) using a surgical scalpel and immersed in a 5% (v/v) sodium hypochlorite solution (Sigma-Aldrich, St. Louis, MO, USA) to facilitate the degradation of organic matter. After this step, the samples underwent multiple washes with distilled water to eliminate any remaining sodium hypochlorite. Subsequently, a solution composed of nitric acid (Sigma-Aldrich, St. Louis, MO, USA) and sulfuric acid (Sigma-Aldrich, St. Louis, MO, USA) in a 1:4 ratio was added, and the reaction was allowed to proceed for 24 h. The BS was then left to settle, followed by repeated washing steps with distilled water until the pH exceeded 6, as verified by using a pH meter. Finally, the BS was dried in an oven at 37 °C until complete dehydration and then sieved to obtain particles with an approximate size of 106 μm.

### Obtention of nBS

The nBS were obtained using a planetary ball mill. A 3.6 g amount of BS and 11 mL of 70% ethanol were placed in a ceramic container with five small and two large zirconium oxide spheres. Milling was carried out at 500 rpm for 2 h, including three 15 min breaks to avoid overheating.

### Solution Preparation

To prepare the electrospun solutions, 10 mL of an 18% w/v solution of PCL in glacial acetic acid, a benign solvent, was mixed with 10, 15, and 20 wt % (as observed in Table 1) of nBS particles and kept under magnetic stirring for 24 h.

**Table 1:** Proportion of nBS for Each Group

Group	PCL (g)	nBS (g)
**PCL**	1.815	-
**10 wt %**	1.815	0.1815
**15 wt %**	1.815	0.2722
**20 wt %**	1.815	0.363

### Electrospun Process

After complete dispersion, the fluids were electrospun in a YFlow 2.2.D-350 electrospinning device, by using an ∼13 kV voltage, 1 mL/h flow rate at 21 °C, and 35% relative humidity. A needle with a 0.8 mm diameter was located 20 cm away from the flat aluminum collector. After processing, membranes obtained (referred to as “M” series) were stored in a desiccator until usage.

### Film Formation

The nBS-containing PCL solutions as described above were casted into films under ambient conditions until complete formation of the flat nonporous composite counterparts (referred to as “F” series).

### Surface Modification

Samples subjected to surface modification by basic hydrolysis are indicated with “m”. Both the M series and F series were immersed in NaOH 5 mol/L for 3 h to modify the wettability of the materials (referred to as “mM” series for membranes and “mF” series for casted films).

### Physicochemical and Thermal Characterization

The morphology of nBS and the surface analysis of the M series and F series were investigated by scanning electron microscopy (SEM) (FE-SEM-EDS; Zeiss Crossbeam 350, Germany) after chrome sputtering using a sputter coater (Q150T, Quorum Technologies, Darmstadt, Germany). The nanofiber diameter distributions were calculated based on the micrographs using the ImageProPlus software (Media Cybernetics, Rockville, MD, USA) for the measurement of 100 repetitions for each experimental group with the statistical test. For the relative quantification of the atomic elements present in the M series and F series, energy dispersive spectroscopy (EDS) analysis was performed at 1000× magnification.

### Contact Angle (CA)

The wettability of the samples was estimated by measuring the contact angle with a goniometer (Ramé-HartCo.; USA), by releasing 4 μL of distilled water (Krüss DSA30, Hamburg, Germany) on the material surface.

### Fourier Transform Infrared (FTIR)

FTIR spectra were obtained using a Thermo Scientific Nicolet 6700 spectrometer, over a range of 500–4000 cm^–1^ with a resolution of 2 cm^–1^ and 64 averaged scans.

### X-ray Diffraction (XRD)

XRD analysis was performed with an X’pert Pro PANalytical diffractometer, at 40 kV 30 mA, in a range of 3–60° in 2θ with a step size of 0.02°.

### Thermogravimetric Analysis (TGA)

The composite thermal degradation was studied by TGA in a TA Auto-MTG Q500 HI-Res instrument (TA Instruments). The scans were carried out from 25 to 800 °C at 10 °C/min, in a nitrogen atmosphere.

### Biomineralization

The biomineralization of membranes and films was assessed by their apatite-forming ability on the surface during immersion in simulated body fluid (SBF) solution for 21 days.^5^ At days 0, 12, and 21, 2 mL of the solution was collected for Ca^2+^ ion stimulation and replaced with fresh media. The Ca^2+^ ion release content was estimated by comparing the element content in the SBF supernatant using X-ray fluorescence (XRF) emission in a PANalytical MiniPal2 equipment with a chromium anode in a nitrogen atmosphere. Scans were performed at 20 kV, 15 μA during 120 s. After immersion, the samples were examined by SEM/EDX after gold sputtering.

### *In Vitro* Studies

The biological response to the membranes was assessed using pre-osteoblast cells (MC3T3-E1; IMBICE, Argentina). Cells were cultured in bottles using DMEM (Serendipia, Argentina) supplemented with 10% FBS (Internegocios, Argentina) and 1% antibiotic–antimycotic solution (penicillin–streptomycin, Serendipia; Argentina) at 37 °C in a humidified atmosphere of 5% CO_2_. They were maintained at subconfluent densities and were passed weekly until use.

Cells in a density of 2.25 × 10^4^ cells cm^–2^ were seeded on top of films, membranes, and a cellular control without any sample seeded on the well, in triplicate. Wells were filled with culture medium after 1 h of cell seeding and incubated for 24 h at 37 °C and 5% CO_2_. Then, cell morphology was observed after fixation and DAPI (Sigma-Aldrich) and phalloidin (Alexa Fluor 488, Invitrogen) staining. Random fields at 20× and 40× magnification were then imaged (Leica DM IL LED, Germany).

### Statistical Analysis

Data related to the manufacturing and characterization results as well as the *in vitro* biological study were presented as means and standard deviations. Initially, the distribution of variables was tested by using the Shapiro–Wilk normality test. For variables that presented a normal distribution, comparisons between groups were performed using analysis of variance (ANOVA, two-way), followed by Tukey’s *post hoc* test. For variables that did not present a normal distribution, the corresponding nonparametric tests were used. The statistical program used was GraphPad Prism version 8.0, and the significance level adopted was 5% (*p* ≤ 0.05).

## Results

The nBS was successfully obtained from the marine sponge species *Dragmacidon reticulatum* (Figure 1). It is possible to observe structures of irregular morphology, with predominantly angular polygonal shapes and clusters formed by smaller granules of less than 0.4 μm. The EDS elemental composition of nBS revealed a predominant composition based on silicon (Si, 80.55%) and oxygen (O, 17.66), with traces of calcium (Ca, 0.16%) and other minor residual mineral compositions from the original biological matrix, as well as from the extraction process.

**Figure 1:**
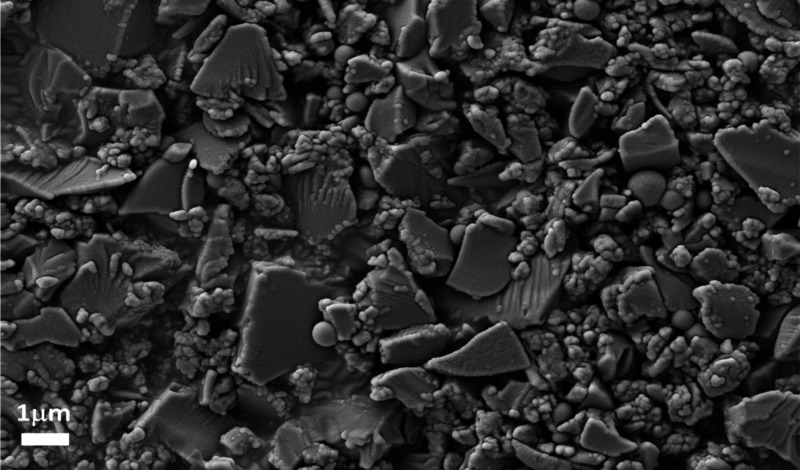
SEM image of the nBS particles.

### Physicochemical Characterization

In Figure 2a, SEM images show the morphology of the M series of PCL membranes containing 0, 10%, 15%, and 20% nBS (M, M10, M15, and M20, respectively). The M series fibers exhibit a homogeneous surface and uniform diameters with no evident defects and interconnected porosity. With increasing filler content, a slight change in surface uniformity was observed, suggesting an initial interaction between the polymer and the nanoparticles that led to surface roughness, slightly larger fiber diameter distributions, and the presence of structural irregularities, including small agglomerations along the fibers (please see white arrows). The surface modification (Figure 2b) did not modify the interconnected porosity but caused certain fiber sticking and left the rough surface of the nanoparticles more exposed, particularly in the composites with higher filler content. An example of a water droplet placed on the nanofibrous mats is shown in Figure 2, in order to easily visualize the increase in surface polarity after the hydrolysis treatment.

**Figure 2:**
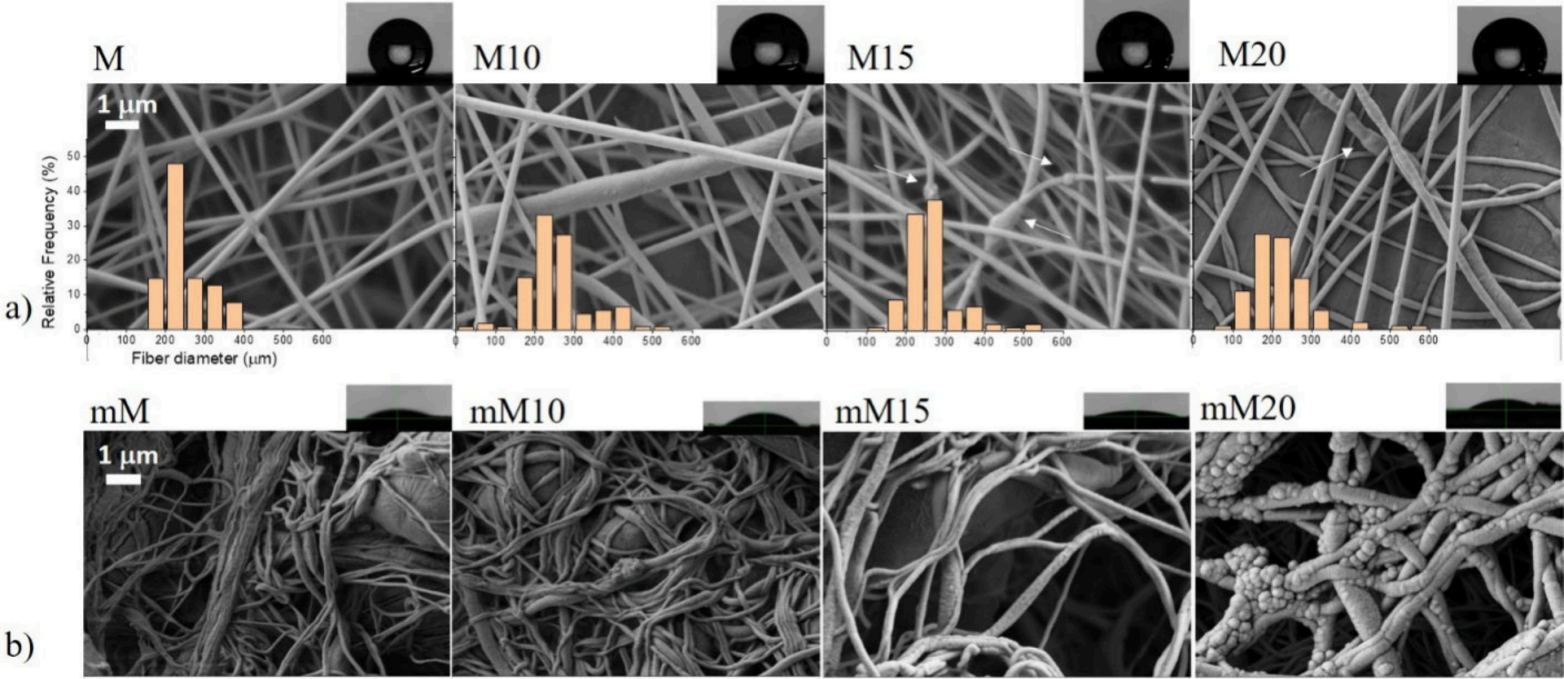
SEM images of M series before (a) and after (b) the surface modification for all compositions. Scale bar = 1 μm. As insets, a water droplet used for contact angle testing is shown.

The compositional analysis obtained from the EDS spectra is summarized in Table 1. In addition to the majority oxygen and carbon, a signal assigned to silicon is detected in the spectra of the composite materials. In the composite films, the Si percentage doubled after the surface modification for F10 and F15 series, but it remained similar for F20. Conversely, the detectable silicon content in the membranes’ surface decreased after the wet hydrolysis in mM10 and mM20 but remained almost constant in the mM15. This opposite trend may be a result of the different impact of the hydrolysis on a continuous film surface than on a submicrometric fibrous membrane with enormous exposed surface area. The erosion caused by the wet hydrolysis acts on the surface of a relatively thick compact film, thus revealing more particles immobilized in the inner part. In the porous membranes, the hydrolysis directly affects a much larger exposed area, and it may be possible to expect some loss of the particles confined to the external part of the very thin fibers, but an overall higher detectable amount. Minor values of calcium were quantifiable only in the M10 and M15. In summary, M15 and F20 showed a constant detectable silicon content before and after the erosion of the surface modification. The inspection of the EDS mapping (Figure 4) reveals an excellent dispersion of Si and Ca within the fibers of mM15, while mF20 shows less content and considerable heterogeneity.

**Figure 3:**
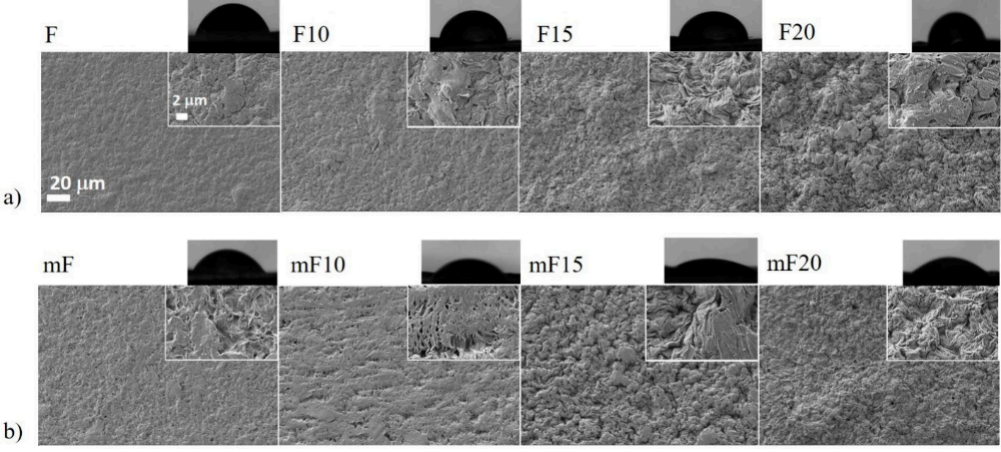
SEM images of the F series with varying nBS (%) content: (a) before and (b) after the surface modification. Zoom-in exhibits the detailed structures. Scale bar: 20 μm. As insets, a water droplet used for contact angle testing is shown for each system.

**Figure 4:**
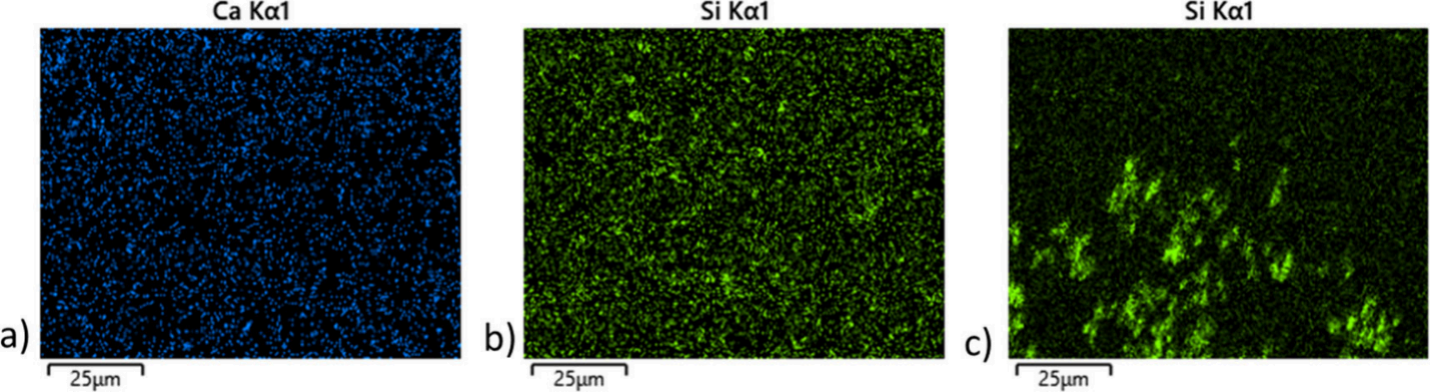
Elemental mapping of calcium in mM15 (a) and silicon in mM15 (b) and mF20 (c).

**2:** Elemental Composition of Different Membranes and Films before and after Surface Modification

	M series							
Element (%)	M	mM	M10	mM10	M15	mM15	M20	mM20
C	61.26	57.88	61.90	53.08	57.18	49.97	58.81	49.00
O	38.69	41.97	36.44	46.08	41.44	48.21	39.51	50.43
Si	-	-	**1.62**	**0.60**	**1.36**	**1.39**	**1.62**	**0.57**
Cl	0.06	0.15	0.03	0.16	-	0.25	0.06	-
Ca	-	-	**0.01**	**0.09**	**0.02**	**0.18**	-	-

	F series							
Element (%)	F0	mF0	F10	mF10	F15	mF15	F20	mF20
C	37.60	47.07	50.18	35.99	44.73	45.63	47.36	49.84
O	62.37	52.84	49.40	63.26	54.84	53.72	51.88	49.43
Si	0.03	0.02	**0.24**	**0.64**	**0.22**	**0.50**	**0.60**	**0.54**
Cl	-	0.08	0.18	0.11	0.20	0.15	0.15	0.18

Casting processing allowed for the acquisition of compact films with structural integrity. The incorporation of nBS filler increased the roughness when compared with neat PCL films (Figure 3a). The wet surface modification increased the roughness even further and clearly changed the film polarity, as seen in Figure 3b.

### CA

Figure 5 shows the measured contact angles of the M series before and after the surface modification. In the modified films, the drop was immediately absorbed before the measurement. As expected, all materials that underwent surface modification led to smaller angles than the original ones.

**Figure 5:**
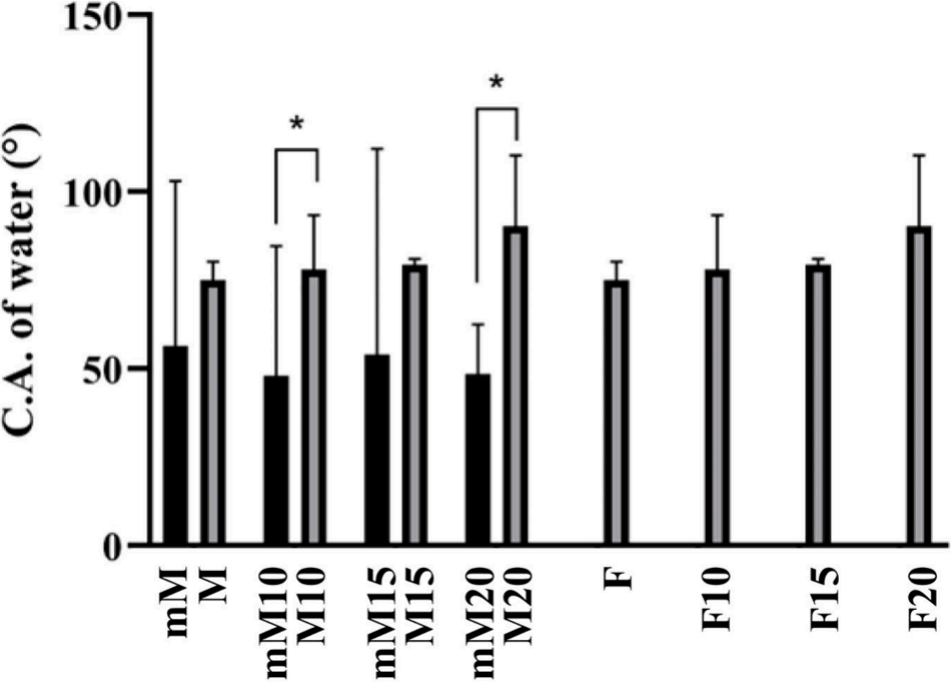
Water contact angle measurements for the nanofibrous mats before and after the surface modification. Statistically significant differences (*) were observed between groups mM10 and M10 and mM20 and M20 (two-way ANOVA, *p* < 0.05).

### FTIR

The typical functional groups detected in the M series are depicted in the spectra in Figure 6. The main peaks are related to the PCL matrix as a majority compound: asymmetric CH_2_ stretching is detected at 2945 cm^–1^, the carbonyl group (C=O) at 1720 cm^–1^, C–H bending at 1292 cm^–1^, asymmetric C–O–C stretching at 1236 cm^–1^, and the band at 1163 cm^–1^ was assigned to C–O and C–C stretching in the amorphous phase. The nBS spectrum shows a peak at 786 cm^–1^ assigned to Si–O or Si–OH stretching and a band at 436 cm^–1^ corresponding to the Si–O–Si group.^14,15^ All the composite M series show this last peak, thus corroborating the correct nBS incorporation within the submicrometer fibers, even after the wet surface modification. Besides, increasing amounts of nBS led to higher intensities at 1090 cm^–1^, thus forming a double peak, while PCL alone shows predominance of the 1105 cm^–1^ peak (arrow in the second rectangle in Figure 6) and just a shoulder at 1090 cm^–1^. This fact may be the consequence of the contribution of the broad band detectable in nBS at 1046 cm^–1^ related to Si–OH bonds and denotes the interaction between the polymer and filler. Finally, the effectiveness of the modification is evident with the appearance of a broad band in the range 3100–3700 cm^–1^ (second arrow), related to the O–H groups formed with the hydrolysis reaction. Similar results were found for the FTIR spectra of the composite films (not shown).

**Figure 6:**
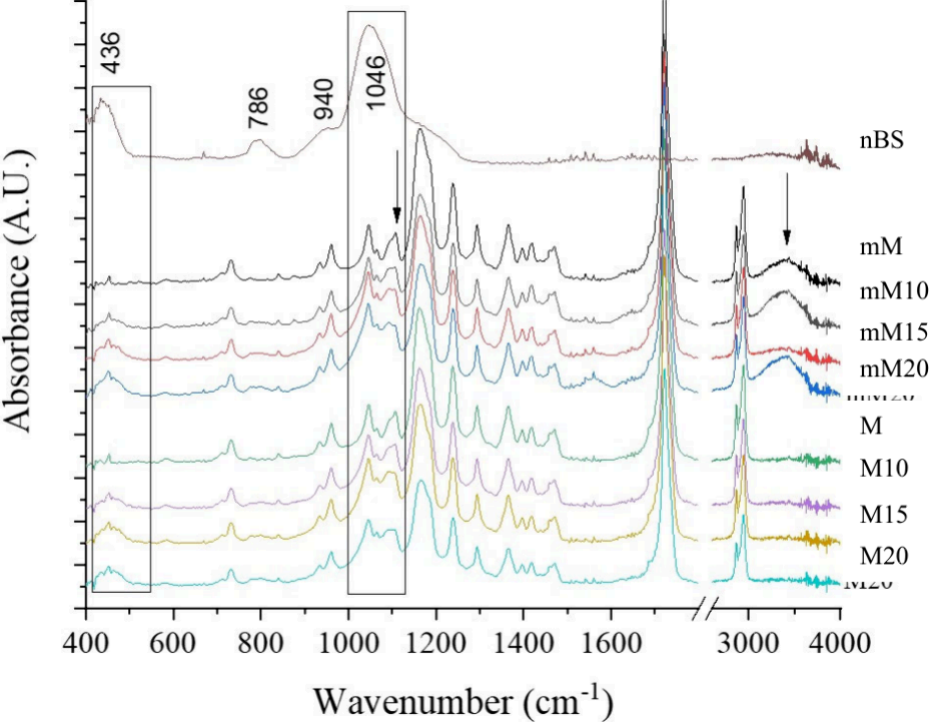
FTIR spectra of nBS and the M series with varying nBS content before and after (“m” series) surface modification.

### TGA

Figure 7 shows the thermal degradation analysis of the M and F series before and after surface modification (M series). For comparison purposes, the degradation curve of the nBS particles was also included in the graphs. As expected, the residual content at 800 °C is minimal for the inorganic nBS particles, with the mass loss being attributed solely to the moisture content of the sample.

**Figure 7:**
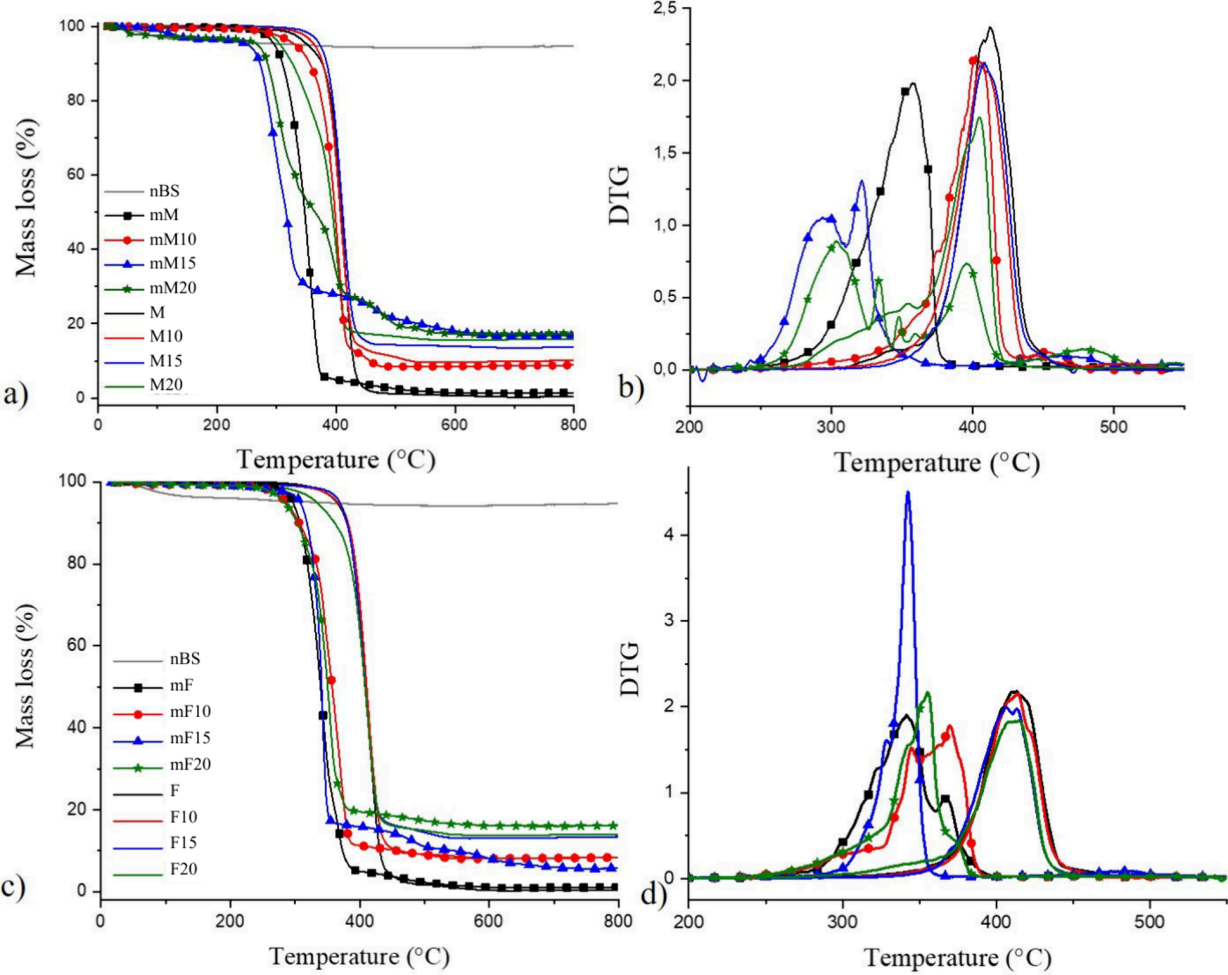
TGA curves of (a) membranes (M) and (c) films (F) based on pure PCL, and PCL composites with 10%, 15%, and 20% nBS content, with (M series) and without surface modification. nBS degradation curve is included for comparison purposes. (b) First derivatives of the DTG for membranes and (d) films with varying compositions.

In the degradation curve (DTG) of the pure electrospun PCL (Figure 7b), two peaks are detected. The maximum degradation rate for the unmodified materials occurs in the same temperature range as the pure PCL film, around 410 °C. However, F20 and M20 start to degrade at lower temperatures.

After surface modification, both the F series and M series showed accelerated degradation at significantly lower temperatures. For pure PCL and PCL with 10% nBS, this acceleration is more pronounced in the films than in the membranes. On the other hand, when the modification is performed on membranes with higher nBS content (15% and 20%), the degradation of the nanofibers is faster than that of the films. Among the modified membranes, the composites with higher BS content (mM15 and mM20) showed faster degradation compared to the unfilled PCL membrane, while there was a delay for mM10. The moisture-corrected residual masses were 9.0%, 12.7%, and 13.6% for mM10, mM15, and mM20, respectively. Interestingly, the final inorganic contents of the modified films were 8.3%, 5.7%, and 16.1%, respectively. The DTG curves of the unmodified materials showed similar shapes and temperature ranges. Nevertheless, significant variations were observed in the degradation mechanisms of the composites, as evidenced by multiple main peaks and distinct events in the curves.

### XRD

XRD analysis of nBS powder showed a predominant amorphous nature for BS, though characteristic peaks were detected at 20.85°, 26.66°, and 50.16° probably related to the hierarchical organization and crystalline structure of *Dragmacidon reticulatum* species spicules.^8^ Figure 8 compares the filler pattern with the XRD profiles of the composite films and M15 and M20. As expected, the peaks at 21.7° and 24.0° are the main ones detected for the composites, related to semicrystalline PCL as the major component of the polymer matrix. However, minor signals are detectable at 26.66°, as well.

**Figure 8:**
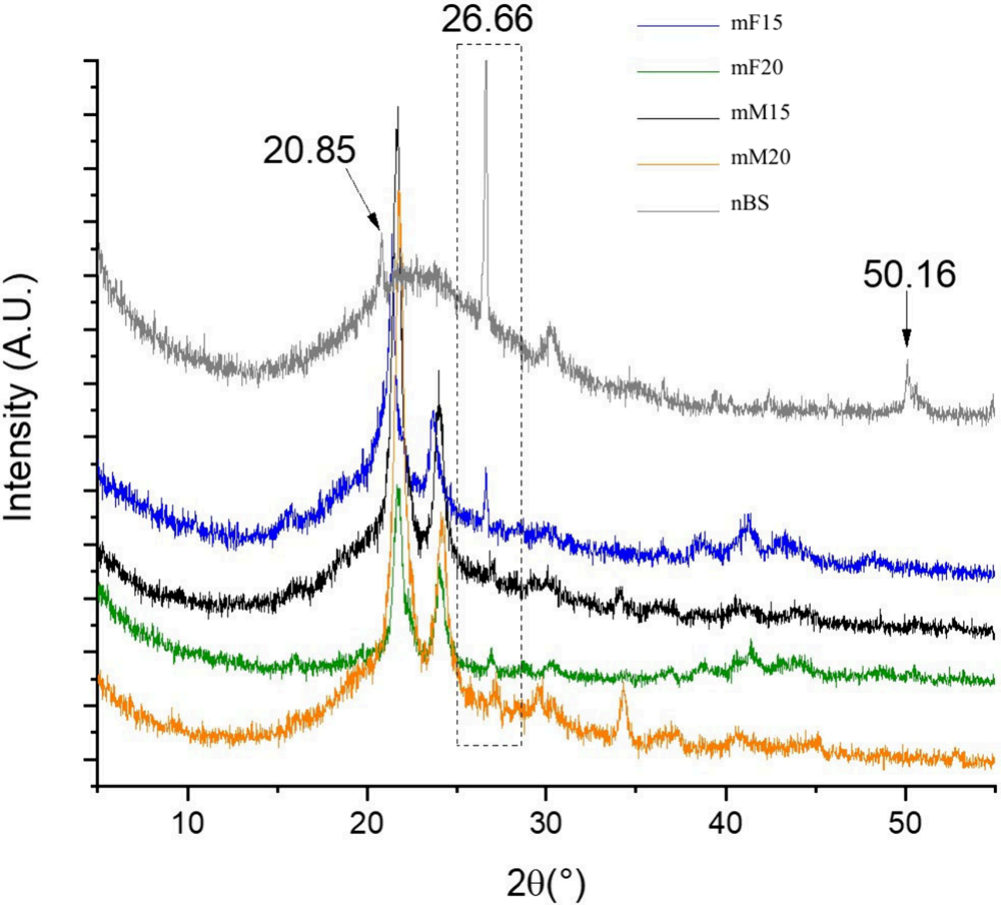
XRD patterns for nBS and the surface-modified composite membranes and films containing 15% and 20% nBS content. The dashed square marks the typical crystal peak of nBS at 26.66°.

### Biomineralization

After 21 days of immersion in SBF, a significant modification in the surface morphology of the samples was observed, particularly in the materials with higher nBS content (Figure 9). In the membranes (a, b), the fibrous structure and interconnected porosity were preserved, with evident deposition of spherical and elongated precipitates on the fibers, being more abundant in the sample M20. In films (c, d), the surface appears more compact and covered by a rough layer, with irregular deposits and globular structures characteristic of the formation of apatite. The sample M20 (d) presents a more homogeneous and thicker coverage, indicating that the higher concentration of nBS favors the nucleation and growth of bioactive minerals. Indeed, the Ca/P molar ratio obtained from EDS elemental mapping was 1.64 for F20, the value more closely related to the 1.67 ratio reported for pure hydroxyapatite, a main component of bone. The elemental mapping obtained from EDS inspection of F20 (a) and M20 (b) samples after SBF immersion shows a quite homogeneous dispersion for the Ca- and P-based deposits, while some silicon agglomerations are detected (Figure S1, Supporting Information).

**Figure 9:**
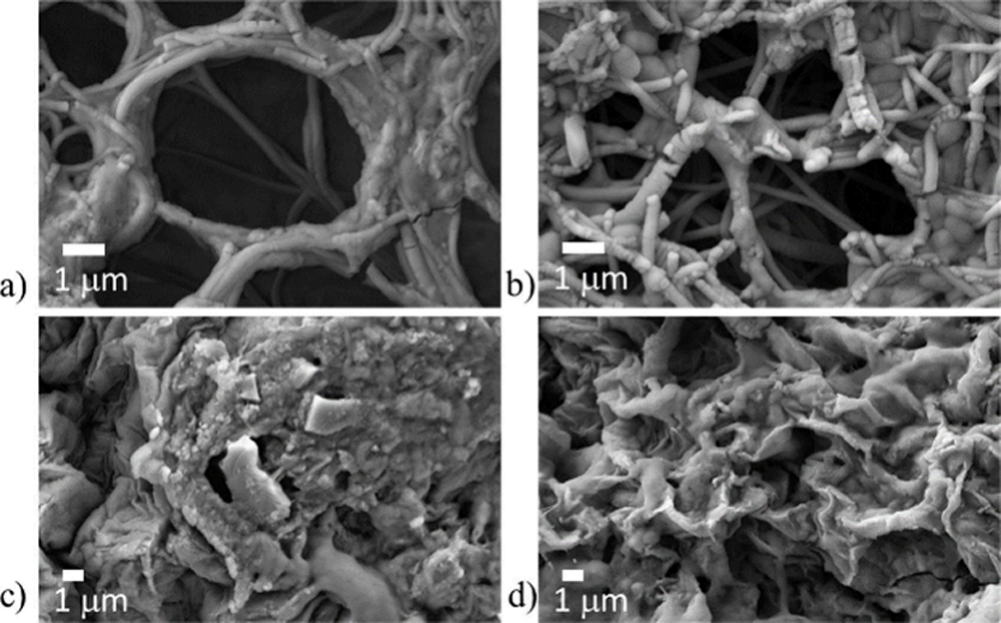
Morphology of the M series (a, b) and F series (c, d) containing 15% and 20% nBS, after 21 days of SBF immersion.

Figure 10 shows a comparative estimation of the Ca^2+^ releases from mM15, mF15, and nBS, obtained from the elemental XRF detection in the SBF supernatant after 10 and 21 days of immersion with respect to the neat SBF media (day 0). Although there are no notable differences at 10 days, after 21 days, it is possible to detect more Ca^2+^ ions in the medium exposed to the nanofibers than to the film or nBS powder, respectively. Nanoscale processing likely helped improve dispersion over a large exposed surface area.

**Figure 10:**
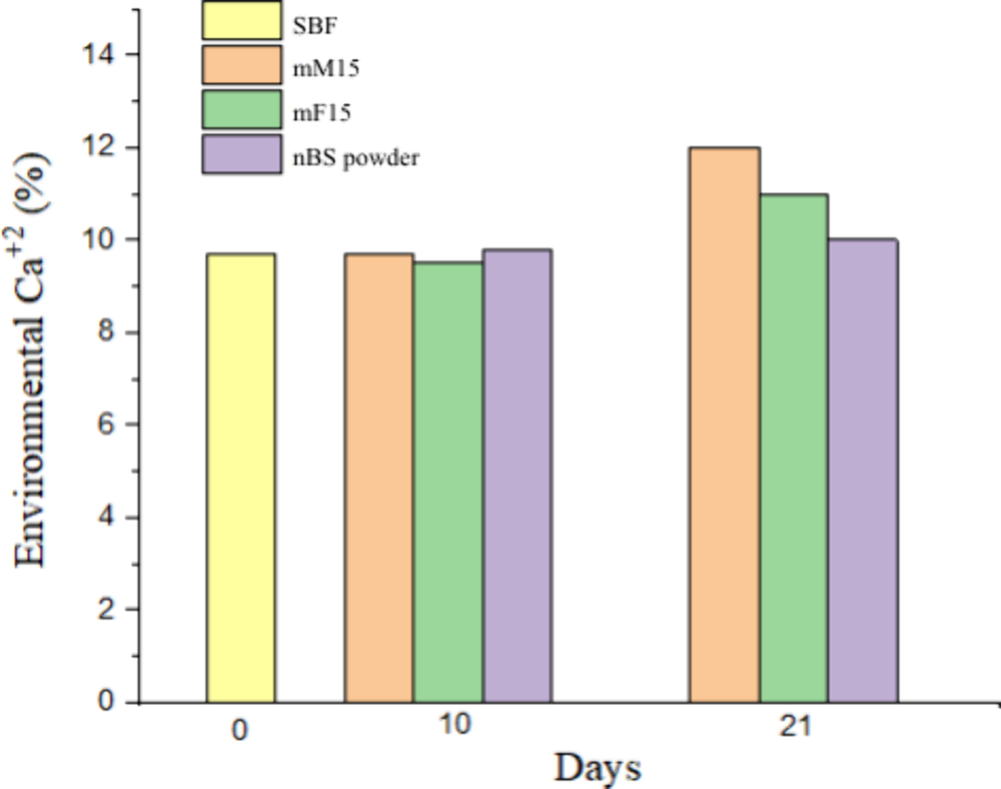
Calcium content (%) detected by XRF in the SBF media (0 days) after 10 and 21 days of immersion of mM15, mF15, and nBS powder. No statistically significant difference between the groups (*p* > 0.05).

### *In Vitro* Cytocompatibility

To assess the biological response of a cell type present in bone, the pre-osteoblasts, an adhesion test was performed on mF, mF15, mM, mM15, and a cellular control without any material (Figure 11). These materials were chosen since mM15 presented the best results, and thus mM, mF, and mF15 were included for comparative purposes. After 24 h of direct contact, cells were detected dispersed onto both types of films and membranes (Figure 11). Through cytoskeleton staining (phalloidin), the cellular morphology was examined. As shown in Figure 11, cells seeded on the plastic bottom of the well (cellular control) show a fully spread morphology. Comparatively, cells on both films (mF and mF15) were spread to a lesser extent than the control, with some cells having polygonal shapes. Cells on the membranes (mM and mM15) presented polygonal morphologies, some of them more spread on the surface and others round-shaped. Moreover, cells on membranes were observed at different focal planes along the *Z*-axis (appearing ‘out of focus’ in Figure 11), suggesting adhesion to the fibers and distribution in the three dimensions. In contrast, in the cellular control and films, cells were confined to a single focal plane, consistent with a 2D adhesion pattern.

**Figure 11:**
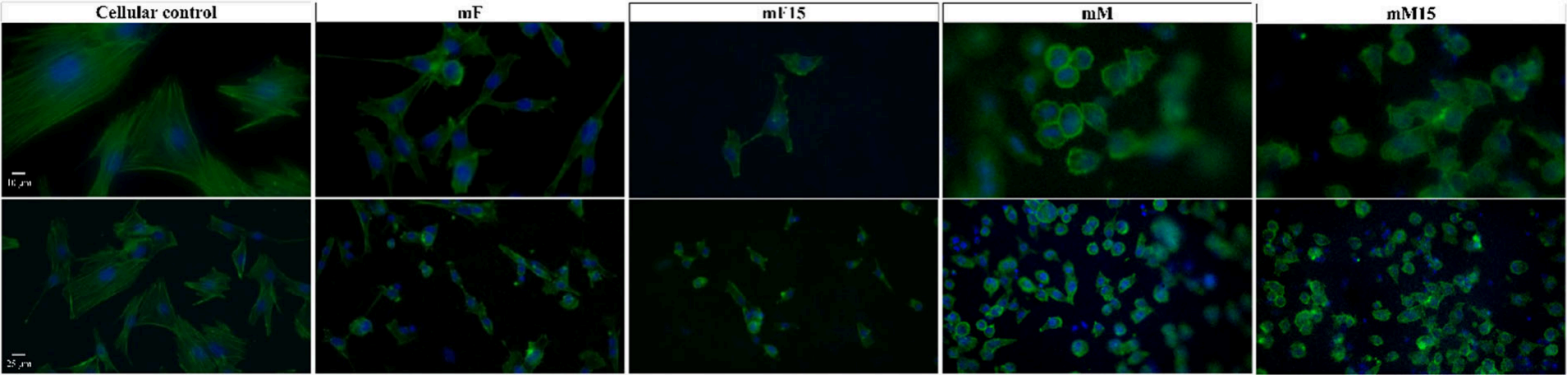
Fluorescence microscopy images of cells cultured for 24 h on a plastic dish (cellular control), mF, mF15, mM, and mM15. The upper images were obtained at 40× magnification (scale bar: 10 μm) and the lower ones at 20× magnification (scale bar: 25 μm).

## Discussion

This study aimed to develop electrospun membranes and casted films with nBS extracted from the marine sponge *Dragmacidon reticulatum*, incorporated into a PCL matrix at relatively large contents (10, 15, and 20 wt %). The physicochemical, surface, morphological, and thermal properties were investigated, as well as the effects of surface modification on biomineralization in simulated body fluid and cell adhesion in *in vitro* tests.

The morphological characterization of the electrospun membranes revealed uniform and interconnected nanofibers in the neat PCL samples. The incorporation of nBS, however, progressively increased fiber surface roughness and broadened the diameter distribution with noticeable surface agglomerates observed at higher concentrations (15% and 20%). This behavior is in agreement with previous studies demonstrating that the inclusion of bioactive fillers, such as silica-based materials, often alters the fiber morphology by interfering with the electrohydrodynamic flow and jet stability during electrospinning, leading to heterogeneous and rougher fiber surfaces.^6,10^

In contrast, the casted films exhibited a compact surface. These differences are expected due to the distinct nature of each processing technique, where electrospinning favors high surface area and porosity, while casting leads to bulk structures. SEM-EDS analysis confirmed the presence of silicon in all composites, indicating the successful incorporation of the BS filler. However, after basic hydrolysis, a decrease in the Si signal was observed in the electrospun membranes, likely due to the partial removal or rearrangement of surface particles caused by the etching process. In contrast, casted films exhibited an increase in surface Si signal following the same treatment, suggesting greater surface exposure of nBS. Nevertheless, despite this superficial increase, the total nBS content in the casted films was significantly lower than that in the electrospun fibers. Moreover, larger agglomerates were observed in the films, indicating a poorer dispersion. By comparison, the electrospun fibers not only incorporated a higher amount of nBS but also achieved a more homogeneous distribution of the nanoparticles throughout the polymer matrix. Larrañaga and Sarasua (2013)^11^ investigated PCL-based films containing 5%, 10%, and 15% bioactive glass and found that the degree of surface enrichment or loss of filler was influenced by both composition and postprocessing conditions. Their findings confirm that particle redistribution or leaching can significantly impact elemental composition at the surface, particularly in materials subjected to hydrolysis or aging treatments. These observations are also consistent with other reports on PCL composites containing bioactive glass, which highlight the interplay between filler concentration, processing method, and the stability of the ceramic–polymer interface.^11,12^

All materials subjected to alkaline surface modification exhibited a significant reduction in the water contact angle, indicating enhanced surface polarity. This change can be attributed to the exposure of hydroxyl groups generated by ester bond cleavage during the alkaline hydrolysis of the PCL backbone, which introduces hydrophilic carboxyl and hydroxyl functionalities at the polymer surface.^13,14^ Such chemical modifications are often accompanied by a simultaneous increase in surface roughness, especially in electrospun membranes, due to their porous and reactive nature. The combined chemical and topographical changes synergistically improve the wettability, which is a key parameter for facilitating protein adsorption and subsequent cell adhesion. Rivero et al. (2019) and Furtos et al. (2017) have shown that hydrophilic and moderately rough surfaces promote better fluid infiltration and cell–material interactions.^6,13^ These properties are particularly desirable in scaffolds for bone regeneration, where hydration, nutrient diffusion, and initial cell anchorage are essential for tissue integration.^4,6,13,15^

FTIR spectra confirmed the characteristic vibrational bands of poly­(ε-caprolactone) in all samples, while the typical Si–O–Si and Si–OH stretching bands were detected in the composites with increasing intensity as the filler concentration rose. Spectral modifications indicate not only the presence of BS in the composites but also potential interactions between the inorganic and organic phases.^8,15^ After basic hydrolysis, a broad absorption band between 3100 and 3700 cm^–1^ appeared, consistent with the formation of hydroxyl-rich surfaces due to the partial degradation of ester bonds.

XRD analysis showed the typical semicrystalline profile of PCL, with diffraction peaks around 21° and 23°, corresponding to the (110) and (200) planes. These peaks were retained in all samples, indicating that the incorporation of nBS and the electrospun processing did not significantly alter the polymer’s crystalline domains. In addition, the amorphous nature of nBS was confirmed by the absence of sharp diffraction peaks related to silica, with the exception of a weak reflection near 26.66°, which may correspond to structural remnants of the marine sponge source material.^8^ These findings align with previous reports stating that amorphous inorganic fillers tend to disperse within the polymer matrix without disrupting the semicrystalline order of PCL, particularly at moderate concentrations.^16^

The thermal degradation of PCL is generally described as a two-step mechanism, involving chain scission through cis-elimination and unzipping depolymerization from the hydroxyl end group of the polymer chain.^11^ The presence of two distinct peaks in the DTG curve of neat electrospun PCL supports this well-established degradation mechanism. When comparing the nonmodified composites, it was observed that both the M series and the F20 began degrading at lower temperatures compared to neat PCL. This behavior is likely due to the excessive filler content, which may concentrate internal stress, promote crack initiation, and reduce the material’s deformation capacity, ultimately leading to earlier thermal degradation.^12,16^ Surface modification of the composites through basic hydrolysis significantly affected their thermal stability. In both morphologies, F series and M series, degradation was accelerated and occurred at lower temperatures. This can be attributed to chain scission promoted by hydrolysis, which generates shorter polymer chains and increases the concentration of hydroxyl end groups.^13,14^ Interestingly, the influence of surface modification varied with both the filler content and morphology. In composites containing 10% nBS, the degradation acceleration was more pronounced in films than in nanofibers. However, for higher filler contents (15% and 20% nBS), the opposite trend was observed: nanofibers degraded more rapidly than their film counterparts This suggests that increasing the surface area of electrospun membranes may increase the accessibility of hydroxide ions, facilitating hydrolysis and subsequent thermal decomposition.^6,12^ This behavior is relevant from a biomedical perspective, as it indicates that the scaffold remains structurally stable under physiological temperature conditions (37 °C). Electrospun PCL scaffolds treated with sodium hydroxide (NaOH) by Bosworth et al. (2019) underwent an optimized protocol that demonstrated improved hydrophilicity and biocompatibility without significantly compromising the material’s mechanical properties. The study revealed that, even after NaOH treatment, the scaffolds maintained their physical integrity, exhibiting only minimal loss of tensile strength.^17^ It can be seen that this balance between surface reactivity and structural preservation is crucial for bone tissue engineering, where temporary mechanical support and progressive bioresorption are necessary for successful tissue regeneration. Among the modified membranes, the thermal stability varied as a function of the filler content. Membranes with 15% and 20% nBS showed accelerated degradation compared to the neat PCL membrane, while the sample mM10 exhibited a slight delay in thermal degradation. This suggests that the incorporation of low concentrations of ceramic nanofillers can enhance thermal stability by forming a char layer and impeding the diffusion of volatile decomposition products, as previously reported for well-dispersed nanocomposites.^12^ In this study, such an improvement was observed only in the 10% nBS modified samples, and the effect was more evident in electrospun membranes (with the DTG peak shifting from 357 to 406 °C) than in films (340 to 370 °C). This enhancement may be related to the electrohydrodynamic processing, which facilitates uniform distribution of the filler within the polymer matrix due to jet stretching and thinning under high voltage.^6,9^ The analysis of the residual inorganic content revealed unexpected results. After surface modification, the final residual masses of the membranes were 9.0%, 12.7%, and 13.6% for mM10, mM15, and mM20, respectively, while in the films, the inorganic content was less consistent: 8.3% for 10%, 5.7% for 15%, and 16.1% for 20% nBS. These discrepancies suggest possible phase separation or agglomeration of nBS particles, particularly in samples with higher filler content. Such inhomogeneities may have hindered the proper dispersion of the ceramic phase, resulting in regions with uneven inorganic distribution. This phenomenon was particularly evident in the 20% nBS series and may explain the more erratic degradation behavior observed in some modified films.^11,16^ Although the DTG curves of nonmodified materials were similar in shape and degradation temperature range, the surface-modified composites displayed significant differences in degradation mechanisms. The presence of multiple peaks in DTG curves indicates the coexistence of several degradation processes, likely resulting from the synergistic effects of filler–polymer interactions and chain disruption caused by hydrolysis. Nanofillers may act as physical barriers or heat dissipators and restrict the polymer chain mobility, thereby influencing the thermal behavior of the material. Furthermore, moisture present on the surface of materials containing Si–O–Si bonds can lead to the formation of SiO^–^ anions, which associate with Na^+^ or Ca^2+^ counterions and catalyze random scission of polymer chains. This phenomenon, previously described in bioglass-containing composites, may have contributed to the accelerated degradation of nanofibers with higher nBS content (>10%) and larger specific surface area.^11,18^

After 21 days of immersion in SBF, both electrospun membranes and casted films exhibited evidence of surface mineralization. In the nanofibrous membranes, particularly those containing 20% nBS, spherical and elongated calcium phosphate deposits were observed on the fiber surface, indicating active biomineralization. Notably, despite the formation of mineral phases, the electrospun membranes preserved their highly porous architecture. This is a critical feature for biomedical applications, as maintaining interconnected porosity during the mineralization process allows the continuous diffusion of biological fluids, oxygen, and nutrients, conditions that are essential for cell infiltration, neotissue formation, and overall bone regeneration. The films showed a more compact apatite layer with globular morphology, and the F20 sample reached a Ca/P molar ratio of 1.64, very close to the stoichiometric value for hydroxyapatite (1.67). However, it is important to highlight that bioactivity involves a dynamic equilibrium between the ions that are mineralized on the surface and those released into the medium, and this balance can vary over time depending on the material’s structure and surface characteristics. Indeed, the comparative XRF analysis of calcium content in the SBF supernatant after 10 and 21 days confirmed this behavior: at day 21, the medium incubated with electrospun membranes (mM15) contained more Ca^2+^ ions than that with casted films (mF15) or the nBS powder. This suggests a higher initial release potential from the nanofibrous structure, likely due to its higher surface area and porosity. However, this increase in ion release does not necessarily reflect lower mineralization but rather reflects the dynamic exchange occurring between the scaffold and the medium. Both the processing method and surface modification strongly influence this balance. Electrospun membranes, by presenting a larger specific surface area and faster ion exchange, may promote a more rapid but transient mineralization process, whereas films may support a more gradual but possibly denser apatite layer formation over time.

Several studies have investigated the bioactivity and cellular responses of BS-based composites, highlighting the importance of material structure, processing, and surface chemistry in modulating osteogenic outcomes. For instance, Müller et al. (2014)^9^ reported that PCL nanofibers incubated with silicatein and silicon precursors displayed BS deposits that supported osteoblastic cell growth and mineralization, though the extent of mineral deposition depended on the scaffold’s surface structure derived from the *in situ* enzymatic fabrication method.^12^ Indeed, Wiens et al. (2010) emphasized that the bioactivity of BS composites is closely related to the availability of silicic acid on the scaffold surface and its enzymatic interactions, which drive mineral nucleation when using this BS formation approach.^3^ In our work, the direct electrospun processing of fluids containing BS led to homogeneously dispersed composite nanofibrous materials, thus avoiding heterogeneous deposits. In general, Wang et al. (2013, 2014) further demonstrated that scaffold porosity and surface modification influence the dynamic release and deposition of calcium and phosphate ions, critical for osteogenic signaling and matrix mineralization^4,18^

Cell adhesion assays with pre-osteoblasts demonstrated that both the neat PCL membranes and the nBS-loaded membrane (mM15) supported early cell attachment after 24 h. Morphological analysis revealed that the adhered cells exhibited characteristics of adhesion in a three-dimensional (polygonal) morphology. Although the incorporation of nBS did not show a notable enhancement of initial adhesion, the surface-modified membranes maintained a stable cell attachment despite the presence of a ceramic phase. These findings suggest that the addition of nBS does not compromise the biological response of the composite and may provide a suitable platform for subsequent stages of cell proliferation and differentiation. The three-dimensional distribution of adhered cells observed in the membranes, in contrast to the two-dimensional adhesion seen on films, supports the hypothesis that the interconnected structure of the biomaterial is essential for supporting cell migration and adhesion within the scaffold. Such behavior highlights the potential of these membranes not only to support surface adhesion but also to enable cell colonization of the scaffold interior, which is a prerequisite for effective tissue integration. In comparison to previous work, differences in BS incorporation strategies, such as physical loading in nanofibers versus *in situ* generation, likely influence the mineralization patterns and cellular responses observed. Gabbai-Armelin et al. (2019) showed cytocompatibility of BS particles from marine sponges without adverse effects on pre-osteoblastic cells, supporting the potential of diverse BS sources and composite structures.^8^ Collectively, these works indicate that the interplay among scaffold architecture, BS concentration, and ion release kinetics is crucial for optimizing osteogenic outcomes, guiding the design of bioactive composites for bone tissue engineering.

Considering that BS is derived from a natural source, a potential concern is the leaching of particles into the surrounding environment. However, previous studies have shown that BS or soluble silica undergoes gradual dissolution into orthosilicic acid (Si­(OH)_4_), a bioavailable, nontoxic form of silicon that can be metabolized and excreted by the body. For instance, orthosilicic acid has been shown to stimulate type I collagen synthesis and osteoblastic differentiation in MG-63 cells, enhancing the expression of ALP and osteocalcin.^19^ Moreover, it was recently reported to inhibit human osteoclast differentiation and bone resorption in RANKL-stimulated cultures.^20^ From an environmental perspective, biosilica is a biogenic and biodegradable material, and thus its potential release poses minimal ecological risk compared with nondegradable synthetic fillers. This aspect further reinforces the safe and bioinspired nature of using biosilica as a filler in polymer-based scaffolds.

Taken together, these findings support the notion that although early stage cell adhesion was not significantly improved, the incorporation of nBS into electrospun PCL membranes results in a biocompatible system with promising potential for bone tissue applications. Future investigations should include longer incubation times, molecular markers of osteogenic differentiation, and *in vivo* assessments to better elucidate the osteoinductive and osteoconductive potential of these nanocomposite membranes.

Tailoring scaffold surfaces at the nanoscale in combination with bioactive cues can lead to significant improvements in osseointegration and host response. The findings of this work demonstrate that integrating nBS into the electrospun process is a highly promising strategy for designing PCL-based scaffolds with tailored surface properties and enhanced bioactivity. The porous nanofibrous architecture contributed to a better dispersion of relatively high filler contents, as evidenced by the characterization results. The composites’ structural features promoted increased mineral deposition and ion exchange rates, which are critical parameters for bone regeneration. Moreover, surface modification via basic hydrolysis significantly improved the hydrophilic character of the composites, potentially favoring cell–material interactions.

## Conclusion

This study demonstrated that electrospinning enabled the homogeneous incorporation of relatively high contents of nBS (10–20 wt %) into PCL matrices, producing porous nanofibrous scaffolds with improved surface area, dispersion, and bioactivity compared with casted films. Surface modification by alkaline hydrolysis further enhanced the hydrophilicity and wettability, creating favorable conditions for protein adsorption and cell adhesion. While early stage adhesion was not significantly affected by the presence of BS, the composites exhibited active biomineralization in simulated body fluid and maintained structural integrity throughout the process, highlighting their suitability as bioactive scaffolds for bone regeneration.

Overall, the synergistic effect of BS incorporation and electrospun processing resulted in composites with tailored physicochemical and biological properties, reinforcing the potential of this strategy for the development of next-generation bone tissue engineering scaffolds. Future work should focus on long-term *in vitro* studies, osteogenic differentiation assays, and *in vivo* evaluations to confirm the osteoinductive and osteoconductive potential of these nanocomposites.

## Supplementary Material



## Notes

Additional experimental details of elemental mapping obtained by EDS inspection of samples F20 and M20 after immersion in SBF (PDF)

## Abbreviations

BS: biosilica
C: carbon
CA: contact angle
Ca: calcium
Ca^2+^: calcium ions
CO_2_: carbon dioxide
DMEM: Dulbecco’s modified Eagle’s medium
DTG: degradation curves
F: casted PCL films
F series: casted films
F10: casted PCL films containing 10% nBS
F15: casted PCL films containing 15% nBS
F20: casted PCL films containing 20% nBS
FBS: fetal bovine serum
FE-SEM-EDS: field emission scanning electron microscopy with energy dispersive X-ray spectroscopy
FTIR: Fourier transform infrared
M: electrospun PCL membranes
M series: electrospun membranes
M10: PCL membranes containing 10% nBS
M15: PCL membranes containing 15% nBS
M20: PCL membranes containing 20% nBS
MC3T3-E1: preosteoblast cells
mF: casted films with NaOH-modified surface
mF10: casted PCL films containing 10% nBS with NaOH-modified surface
mF15: casted PCL films containing 15% nBS with NaOH-modified surface
mF2: casted PCL films containing 20% nBS with NaOH-modified surface
mM: electrospun membranes with NaOH-modified surface
mM10: PCL membranes containing 10% nBS with NaOH-modified surface
mM15: PCL membranes containing 15% nBS with NaOH-modified surface
mM20: PCL membranes containing 20% nBS with NaOH-modified surface
NaOH: sodium hydroxide
nBS: biosilica nanoparticles
O: oxygen
PCL: poly­(ε-caprolactone)
PLA: polylactic acid
SBF: simulated body fluid
Si: silicon
SISBio: Sistema de Autorização e Informação em Biodiversidade
SISGen: Sistema Nacional de Gestão do Patrimônio Genético e do Conhecimento Tradicional Associado
TEOS: tetraethyl orthosilicate
TGA: thermogravimetric analysis
XRD: X-ray diffraction
XRF: X-ray fluorescence.
