# Supplementary material for: Electrospun Polycaprolactone Scaffolds with Marine-Derived Biosilica Nanoparticles (Dragmacidon reticulatum) for Bone Tissue Engineering Applications
Source: ACS Omega. 2025 Nov 10;10(46):56171–82. doi: 10.1021/acsomega.5c07836 (PMC12658661; doi:10.1021/acsomega.5c07836)
Supplement: Supplementary file 1 [file ao5c07836_si_001.pdf]

ELECTROSPUN POLYCAPROLACTONE SCAFFOLDS WITH MARINE-DERIVED BIOSILICA NANOPARTICLES (*DRAGMACIDON RETICULATUM*) FOR BONE TISSUE ENGINEERING APPLICATIONS

Giovanna do Espirito Santo[1,2,4], *Julieta Leticia Merlo*[2], *Pablo Botta*[3], *Ana Cláudia Muniz Rennó*[1*], *Guadalupe Rivero*[3,4].

[1] Department of Biosciences, Federal University of São Paulo (UNIFESP), 136 Silva Jardim Street, Santos, SP, 11015-020, Brazil.

[2] Applied Electrochemistry Area, Materials Science and Technology Research Institute (INTEMA), CONICET-University of Mar del Plata, Colon 10850, 7600 Mar del Plata, Argentina.

[3] Ceramics Area, INTEMA, CONICET-University of Mar del Plata, Colon 10850, 7600 Mar del Plata, Argentina.

[4] Biomedical Polymers Area, INTEMA, CONICET - University of Mar del Plata, Colon 10850, 7600 Mar del Plata, Argentina.

* Corresponding author

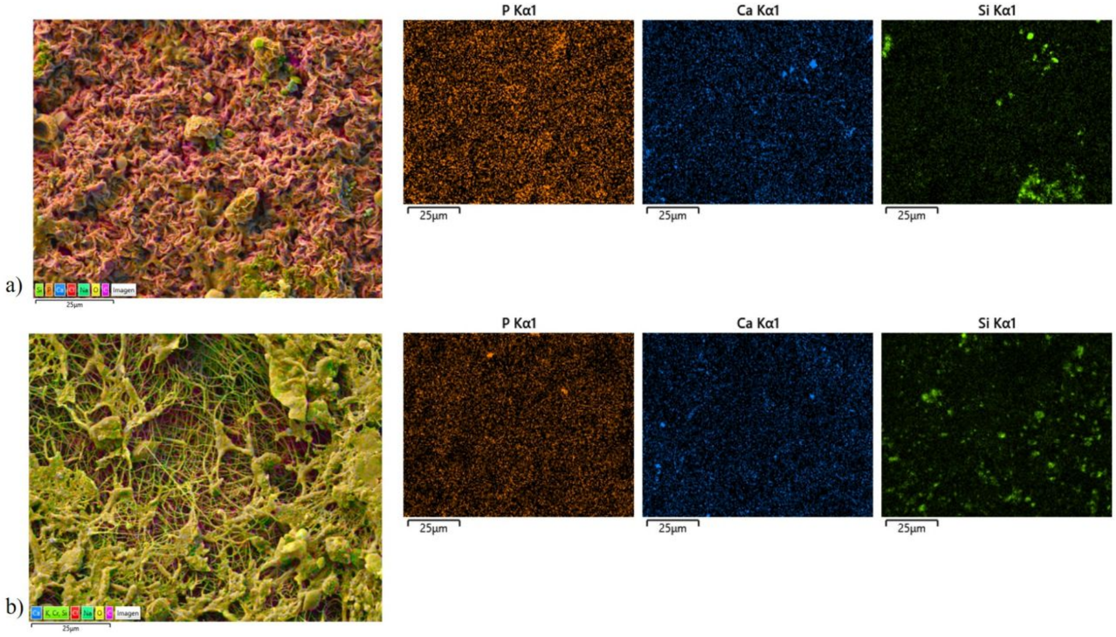

S1. Elemental mapping obtained from EDS inspection of F20 (a) and M20 (b) samples after SBF immersion.
